# Evaluating the need for Epstein-Barr virus DNAemia monitoring in liver transplant recipients in India

**DOI:** 10.1128/spectrum.01862-25

**Published:** 2026-01-06

**Authors:** Reshu Agarwal, Snigdha Purohit, Rahul Garg, Guresh Kumar, Rajeev Khanna, Viniyendra Pamecha, Ekta Gupta

**Affiliations:** 1 Department of Clinical Virology, Institute of Liver and Biliary Sciences 80402, New Delhi, India; 2 Department of Biostatistics, Institute of Liver and Biliary Sciences 80402, New Delhi, India; 3 Department of Pediatric Hepatology, Institute of Liver and Biliary Sciences 80402, New Delhi, India; 4 Department of Hepato-Pancreato-Biliary Surgery, Institute of Liver and Biliary Sciences 80402, New Delhi, India; Mahidol University, Bangkok, Thailand

**Keywords:** liver transplant, EBV DNAemia, PTLD

## Abstract

Epstein-Barr virus (EBV) DNAemia poses a significant risk to transplant recipients, leading to Post-transplant lymphoproliferative disorder (PTLD). This study aims to determine the occurrence of EBV DNAemia among liver transplant (LT) recipients and analyze its association with various clinical parameters. Retrospective data search on 801 patients who underwent LT from January 2015 to December 2024 was performed. Of these, 257 recipients with available EBV DNA test records post-transplant were included and divided into EBV DNAemia and non-EBV DNAemia group. Various pre-transplant (age, MELD/PELD score, transplant type, EBV/CMV serostatus), transplant (cold/warm ischemia time, blood transfused), and post-transplant factors (EBV DNAemia, CMV infection, rejection, and immunosuppressant) were compared in both groups using univariate and multivariate analysis. Out of 257 cases, 138 (53.7%) were adults with a median age of 29 (IQR: 4.5–47) years. EBV DNAemia group included 50 (19.5%) cases with median age 2 (IQR: 1–5) years, majority (56%) classified as high-risk. The median time of EBV DNAemia detection since LT was 319 (IQR: 190–732) days with median viral load of 3.33 (IQR: 2.85–3.80) log_10_ copies/mL. PTLD developed in three cases (both high/intermediate risk). Non-EBV DNAemia group included 207 (80.5%) cases, with median age of 36 (IQR: 11–49) years, primarily belonging to intermediate risk. Age and pre-transplant EBV serostatus were found to be associated with EBV DNAemia on multivariate analysis. Routine monitoring of EBV DNAemia in both adult and pediatric LT recipients, regardless of pre-transplant serostatus, is crucial for early detection and management of EBV DNAemia/associated complications.

**IMPORTANCE:**

There is a significant lack of comprehensive studies on the prevalence and clinical impact of EBV DNAemia among liver transplant (LT) recipients in India. The absence of standardized monitoring and management protocols across Indian transplant centers further adds to inconsistencies in clinical practices, leading to challenges in early detection and intervention. Existing research primarily focuses on the high-risk pediatric renal transplant recipients with limited data available for adult LT recipients. This study aims to address these gaps by providing crucial insights into EBV DNAemia among Indian LT recipients and its association with various clinical parameters.

## INTRODUCTION

Advances in immunosuppressive therapy have significantly improved the overall survival of solid organ transplant (SOT) recipients but have also increased the risk of opportunistic infections including Epstein-Barr virus (EBV) (1, 2). EBV infects and establishes lifelong latency in B lymphocytes following primary infection and is under constant immune surveillance (3). However, post-transplant immunosuppression can lead to EBV DNAemia, through either viral reactivation or primary infection. EBV DNAemia can subsequently increase the risk of post-transplant lymphoproliferative disorder (PTLD) associated with high morbidity and mortality (4).

Various risk factors including age, pre-transplant EBV/cytomegalovirus (CMV) serostatus, and type of immunosuppressive therapy have been associated with occurrence of EBV DNAemia/PTLD (2, 5–7). Since PTLD often follows high EBV DNAemia levels, EBV viral load monitoring has become widespread in transplant recipients. However, clinical evidence supporting the utility of EBV DNAemia monitoring among SOT recipients remains limited (8). Current guidelines recommend EBV DNAemia monitoring primarily in high-risk (D+R−) recipients during the first year post-transplantation, but follow-up strategies beyond this period remain unclear (6). Additionally, a clinically significant EBV DNAemia threshold for predicting PTLD risk has not been established (6, 9, 10). Despite these challenges, current preemptive strategies rely on serial EBV DNAemia monitoring and reduction in immunosuppression (RIS) for minimizing the risk of EBV-related complications, including PTLD.

In India, where EBV seroprevalence exceeds 90% by age five, most transplant recipients fall into the intermediate-risk category (D+R+/D−R+) (11). The lack of standardized protocols for EBV monitoring and comprehensive studies on the clinical impact of EBV DNAemia among liver transplant (LT) recipients further contributes to variability in clinical practices. Existing research primarily focuses on pediatric renal transplant recipients with limited data available on adult LT recipients (12–15). Moreover, the role of immunosuppressive therapy, viral co-infections, and other clinical parameters on the occurrence of EBV DNAemia in the Indian population remains inadequately explored. Therefore, the present study aims to determine the occurrence of EBV DNAemia in both pediatric and adult LT recipients and analyze its association with various clinical parameters.

## MATERIALS AND METHODS

### Study design and study population

A retrospective data search on 801 cases that underwent LT at our tertiary care institute from 1 January 2015 to 31 December 2024 was performed. Among these, EBV DNA test records were available for 257 (32%) recipients who were included in the study. At our center, there is no well-defined post-transplant EBV monitoring protocol for LT recipients and is entirely guided by clinical judgment. EBV monitoring is primarily offered to high-risk pediatric patients based on clinical judgment, but not at predefined intervals. In the present study, we observed that once EBV DNAemia was detected, follow-up testing was generally requested every 1 to 3 months. Based on EBV DNA test results, the study population was classified into two groups: EBV DNAemia and non-EBV DNAemia (see Fig. S1 at https://doi.org/10.5281/zenodo.17986044). At our center, the standard immunosuppression protocol consisted of intra-operative administration of corticosteroids (methylprednisolone) during the anhepatic phase, followed by tapering over 5 days. Maintenance immunosuppression comprised a combination regimen of a calcineurin inhibitor (tacrolimus), an anti-metabolite (mycophenolate mofetil), and corticosteroids (prednisolone). None of the recipients received induction therapy with anti-thymocyte globulin, anti-CD3 monoclonal antibodies, or IL-2 receptor antagonist basiliximab. Comprehensive clinical details and follow-up data for all cases were collected from the hospital information system (HIS), extending up to 31 May 2025. None of the enrolled cases underwent re-transplantation during the follow-up period. Only biopsy-proven graft rejection reported at any time post-transplant was recorded. Information on the maintenance immunosuppressive therapy at the time of EBV DNA test request was noted and was further classified into three-drug (calcineurin inhibitor + anti-metabolite + corticosteroids) or two-drug regimens (calcineurin inhibitor + corticosteroids or calcineurin inhibitor + anti-metabolite). The cases with detected EBV DNAemia were managed by RIS with or without the addition of antiviral (valganciclovir or ganciclovir) based on clinical decisions. The duration of antiviral therapy was determined on a case-by-case basis by the clinicians, as there is no established treatment for EBV.

For the purpose of this study, RIS was defined as either withholding a drug or lowering its dosage. The case was considered EBV-associated PTLD based on histopathological confirmation of PTLD along with the presence of EBV DNAemia. Clinical details so obtained from HIS were categorized into: pre-transplant (before the surgery), transplant (during the surgery), and post-transplant (after the surgery) factors and were compared between EBV DNAemia and non-EBV DNAemia group (see Fig. S1 at https://doi.org/10.5281/zenodo.17986044).

### Virological markers

Pre-transplant risk stratification for EBV was based on anti-EBV viral capsid antigen (VCA)/ early antigen (EA) IgG using Vidas enzyme immunoassay (bioMérieux) in all recipients (R) and donors (D). Similarly, pre-transplant CMV risk stratification was based on anti-CMV IgG using Vidas enzyme immunoassay (bioMérieux). Based on the results, cases were categorized into high-risk (D+R−), intermediate-risk (D+R+/D−R+), and low-risk (D−R−) groups.

EBV DNA was detected using Artus EBV QS-RGQ on Rotor-Gene Q PCR System (QIAGEN GmbH, QIAGEN Strasse 1,D-40724 Hilden). The linear range of the assay was 2.49–7 log_10_ copies/mL with the lower limit of detection being 2.19 log_10_copies/mL. EBV DNAemia was defined as the detection of EBV DNA >2.30 log_10_ copies/mL in plasma specimen. For the purpose of this study, sustained viremia was defined as persistence of EBV DNA >2.30 log_₁₀_ copies/mL for at least 60 days, as documented on three consecutive specimens, each collected at a minimum interval of 30 days. Based on EBV DNA test results, the recipients were further classified into two groups: EBV DNAemia and non-EBV DNAemia.

### Statistical analysis 

Statistical analysis was done using SPSS version 28 (SPSS Inc., Chicago, IL, USA). Continuous variables were summarized using mean ± standard deviation or in median and interquartile range (IQR) where applicable. Categorical variables were presented as percentages. Comparison of two continuous variables was done by Student *t*-test or Mann-Whitney test while categorical variables by Pearson’s chi-square test. Binary logistic regression (both univariate and multivariate) was employed to identify predictors of EBV DNAemia development post-transplant. Variables with *P* < 0.05 in the univariate analysis were included in the multivariate analysis. All statistical tools were two-tailed, and level (*P*) of <0.05 was considered significant.

## RESULTS

### Baseline characteristics of enrolled cases (*n* = 257)

Records of clinician-guided, EBV DNA test requests were available for 257 recipients, each undergoing the testing at least once during post-transplant follow-up. Of these, 116 (45.1%) recipients had EBV DNA test requested at multiple time points. The median age was 29 (IQR: 4.5–47) years with male preponderance (Table 1). Adult recipients constituted the majority (53.7%, *n* = 138) of cases. Most recipients (79.7%, *n* = 205) had prior exposure to EBV, placing them at intermediate risk (D+R+) for EBV post-transplantation. Overall, graft rejection was seen in 94 (36.5%) cases, with none progressing to graft failure. Based on EBV DNA test results, the recipients were classified into two groups: EBV DNAemia (*n* = 50) and non-EBV DNAemia (*n* = 207). Only three cases (1.2%) developed EBV-associated PTLD, all belonging to the EBV DNAemia group. The overall mortality was recorded in 37 (14.4%) cases.

**TABLE 1:** Baseline characteristics of study group (*n* = 257)

Parameter	Number
Age in years, median (IQR)	29 (4.5–47)
Male:female	2.9:1
Population, *n* (**%**)	
Pediatric	119 (46.3%)
Adult	138 (53.7%)
Type of transplant, *n* (**%**)	
Living donor liver transplant	223 (86.8%)
Deceased donor liver transplant	34 (13.2%)
Indication for liver transplant in pediatrics, *n* (**%**)	
Biliary atresia	47 (39.5%)
Acute liver failure	30 (25.2%)
Progressive familial intrahepatic cholestasis	09 (7.6%)
Wilson disease	08 (6.7%)
Others^*a*^	25 (21%)
Indication for liver transplant in adults, *n* (**%**)	
Alcoholic liver disease	37 (26.8%)
Chronic hepatitis B and C	28 (20.3%)
Nonalcoholic steatohepatitis	25 (18.1%)
Cryptogenic	23 (16.7%)
Others^*b*^	25 (18.1%)
MELD/PELD score wherever applicable, median (IQR)	25 (17.1–30.5)
EBV risk stratification based on pre-transplant EBV IgG, *n* (**%**)	
High (D+R−)	52 (20.3%)
Intermediate (D+R+)	205 (79.7%)
EBV DNA, *n* (**%**)	
EBV DNAemia detected	50 (19.5%)
EBV DNAemia not detected	207 (80.5%)
Cases with graft rejection at any point following transplant, *n* (**%**)	94 (36.5%)
Development of PTLD, *n* (**%**)^*c*^	03 (1.2%)
Mortality, *n* (**%**)	37 (14.4%)

^*a*^ Others include autoimmune hepatitis (*n *= 5), cryptogenic (*n *= 4), metabolic liver disease (*n *= 3), primary sclerosing cholangitis (*n *= 3), overlap syndrome (*n *= 2), neonatal sclerosing cholangitis (*n *= 1), secondary sclerosing cholangitis (*n *= 1), glycogen storage disorder (*n *= 1),hepatitis B virus infection (*n *= 1), Alagille syndrome (*n *= 1), hepatocellular carcinoma (*n *= 1), hepatoblastoma (*n *= 1), and congenital porto-systemic shunt (*n *= 1).

^*b*^ Others include autoimmune hepatitis (*n *= 7), acute liver failure (*n *= 6), overlap syndrome (*n *= 3), primary sclerosing cholangitis (*n *= 2), epitheloid hemangio-endothelioma of liver (*n *= 2), primary biliary cholangitis (*n *= 1), progressive familial intrahepatic cholestasis (*n *= 1), Wilson disease (*n *= 1), Budd Chiari syndrome (*n *= 1), and hepatic venous outflow tract obstruction (*n *= 1).

^*c*^ PTLD, post-transplant lymphoproliferative disorder.

### Demographic and clinical details of EBV DNAemia group (*n* = 50)

Table 2 describes the demographic and clinical details of cases belonging to EBV DNAemia group (*n* = 50, 19.5%). EBV DNAemia was detected primarily in the pediatric population with a median age of 2 (IQR: 1–5) years. The majority of recipients (56%, *n* = 28) had never been exposed to EBV, placing them in the high-risk category. Overall, 58% (*n* = 29) of cases developed EBV DNAemia within the first year after transplantation, with the highest frequency (32%, *n* = 16) noted during the 7–12 months period and median time of 319 (IQR: 190–732) days. Earlier detection (245 [IQR: 136–441] vs 713 [IQR: 247–1,290] days, *P* = 0.004)with higher viral load (3.39 [IQR: 2.99–3.82] vs 3.19 [IQR: 2.78–3.80] log_10_ copies/mL, *P* = 0.09) was observed among high-risk when compared to intermediate-risk category.

**TABLE 2:** Demographic and clinical details of cases belonging to the EBV DNAemia group (*n* = 50)

Parameter	Number
Age in years, median (IQR)	2 (1–5)
Male:female	1.4:1
Population, *n* (**%**)	
Pediatric	42 (84%)
Adult	08 (16%)
Type of transplant, *n* (**%**)	
Living donor liver transplant	49 (98%)
Deceased donor liver transplant	01 (2%)
Indication for liver transplant in pediatrics, *n* (**%**)	
Biliary atresia	25 (59.5%)
Acute liver failure	07 (16.7%)
Others^*a*^	10 (23.8%)
Indication for liver transplant in adults, *n* (**%**)	
Alcoholic liver disease	03 (37.5%)
Chronic hepatitis B and C	03 (37.5%)
Cryptogenic	01 (12.5%)
Hepatic venous outflow tract obstruction	01 (12.5%)
EBV risk stratification based on pre-transplant EBV IgG, *n* (**%**)	
High (D+R−)	28 (56%)
Intermediate (D+R+)	22 (44%)
EBV viral load (log_10_ copies/mL), median (IQR)	3.33 (2.85–3.80)
Post-transplant duration from the first detection of EBV DNAemia, *n* (**%**)	
<1 month	None
1–6 months	13 (26%)
7–12 months	16 (32%)
13–18 months	04 (8%)
19–24 months	06 (12%)
>24 months	11 (22%)
Concurrent occurrence of CMV infection, *n* (**%**)	08 (16%)

^*a*^ Others include progressive familial intrahepatic cholestasis (*n *= 3), autoimmune hepatitis (*n *= 1), hepatoblastoma (*n *= 1), congenital porto-systemic shunt (*n *= 1), cryptogenic (*n *= 1), metabolic liver disease (*n *= 1), glycogen storage disorder (*n *= 1), and Alagille syndrome (*n *= 1).

Of the 50 cases, 34 (68%) recipients were on a three-drug regimen, while the remaining 16 (32%) were on a two-drug regimen. EBV DNAemia was managed with RIS alone in 36 cases (72%) and with a combination of RIS and antiviral therapy (valganciclovir) in 14 cases (28%), irrespective of EBV risk stratification. The median viral load did not differ significantly between cases managed with antivirals and those without (3.17 [IQR: 2.72–3.80] vs 3.34 [IQR: 2.95–3.77] log₁₀ copies/mL, *P* = 0.63). Similarly, no significant difference was observed in the median time from LT to EBV DNAemia detection between the two groups (321 [IQR: 130–591] vs 299 [IQR: 200–738] days, *P* = 0.83). Notably, PTLD developed in both groups, irrespective of antiviral use (*P* = 0.65). Overall mortality was recorded in 6 (12%) cases.

Concurrent CMV infection (defined as detection of CMV DNA >2.7 log10 IU/mL in plasma specimen within 7 days of EBV DNAemia) was observed in 8 (16%) cases. These cases were treated for CMV infection using valganciclovir or ganciclovir, adjusted based on renal function. Treatment continued until CMV DNA was undetectable or below the defined threshold for at least two consecutive weeks.

### Follow-up monitoring  

Follow-up records for EBV DNA test were available for 46 out of 50 cases (92%). The sustained EBV DNAemia (as described in methodology) was recorded in 33 cases (71.7%, 33/46). The median viral load was 3.38 (IQR: 2.95–3.81) log_10_ copies/mL and the median time since LT to detection of EBV DNAemia was 268 (IQR: 171–589) days. Of the 33 cases, 23 (69.7%) consistently tested positive during follow-up, with a median duration of DNAemia positivity of 456 (IQR: 134–1,078) days from the initial detection and subsequently leading to EBV-associated PTLD in three (described below). Other 5 (15.1%) cases with sustained EBV DNAemia tested negative during further follow-up.

The remaining 5 (15.1%, 5/33) cases had intermittent EBV-positive results on one or more occasions, which were not consecutive and may or may not turned negative on follow-up testing.

### Development of PTLD (*n* = 3)

In this cohort, the incidence of PTLD was low, with only three cases reported, representing 1.2% of the study population. PTLD occurred in both pediatric and adult population, with two belonging to intermediate and one to high-risk category for EBV post-transplant. All cases had sustained EBV DNAemia that consistently tested positive on follow-up testing. EBV DNAemia in case 1 was managed with RIS and antiviral therapy (valganciclovir) added at various intervals during follow-up. Cases 2 and 3 were managed with RIS alone. Following PTLD development, all cases were managed with the combination of RIS and chemotherapy (rituximab, cyclophosphamide, doxorubicin, vincristine, prednisolone [R-CHOP]). This resulted in the resolution of DNAemia in Cases 1 and 2, but not in Case 3, as the patient succumbed to the illness during treatment. Detailed information regarding these cases is provided in Table 3.

**TABLE 3:** Clinical details of PTLD cases (*n* = 3)^*a*^

Clinical parameter	Case 1	Case 2	Case 3
Age at time of transplant/sex	2 years/male	4 years/male	55 years/female
Indication for transplant	Biliary atresia	Acute liver failure	Hepatic venous outflow tract obstruction
EBV risk stratification based on pre-transplant EBV IgG	High (D+R−)	Intermediate (D+R+)	Intermediate (D+R+)
CMV risk stratification based on pre-transplant CMV IgG	Intermediate (D+R+)	Intermediate (D+R+)	Intermediate (D+R+)
Time since transplant to first detection of EBV DNAemia	11 months	7 months	7 months
Time since first EBV DNAemia detection to PTLD diagnosis	1 year 10 months	2 years 11 months	1 month
Time since transplant to PTLD diagnosis	2 years 9 months	3 years 6 months	8 months
Peak EBV viral load (log _10_ copies/mL)	5.3	5.4	6.1
EBV viral load (log_10_ copies/mL), median (IQR)	3.83 (3.53–4.12)	3.25 (2.93–3.77)	6.07 (5.88–6.15)
Management for EBV DNAemia before PTLD diagnosis	RIS and valganciclovir	RIS	RIS
CMV infection before PTLD diagnosis	Yes	No	No
Clinical presentation	Fever, swelling in right cheek × 1 month	Abdominal pain, fever, loss of appetite, weight loss × 3 months	Weight loss and decreased appetite × 1 month
Pathological finding	Burkitt lymphoma-stage 4	Burkitt lymphoma-stage 3	Diffuse large B-cell lymphoma
Involved PTLD sites	Right maxillary sinus, right retro-orbital region, right sub-mandibular region, right kidney, right iliac fossa, marrow/skeletal sites, cervical lymph nodes, abdominal and messentric lymph nodes	Jejunum, right hypochondrium, right lumbar region, bilateral cervical, anterior supra diaphragmatic, abdominal and messentric lymph nodes	Caudate lobe and adjoining segments IVA and IVB, omental lesions, axial and proximal appendicular skeletal marrow
Treatment	8 pulses of rituximab/4 cycles of CHOP	8 pulses of rituximab/4 cycles of CHOP	1 pulse of rituximab/1 cycle of CHOP
Outcome	Died	Alive	Died

^*a*^ CHOP, cyclophosphamide, doxorubicin, vincristine, prednisolone; RIS, reduction in immnosuppression.

### Correlation of clinical parameters with occurrence of EBV DNAemia

The clinical parameters were categorized into pre-transplant (before surgery), transplant (during surgery), and post-transplant (after surgery) factors. These parameters were compared between EBV DNAemia (*n* = 50) and non-EBV DNAemia (*n* = 207) groups.

Among the pre-transplant factors, significant differences were observed in age, gender, type of transplant, and pre-transplant EBV/CMV serostatus between the two groups. EBV DNAemia group comprised a significantly younger population (median age: 2 [IQR: 1–5] years) compared to non-EBV DNAemia group (median age: 36 [IQR: 11–49] years; *P* < 0.001). A significant association was observed between pre-transplant EBV/CMV serostatus and EBV DNAemia, with a higher proportion of high-risk cases in the EBV DNAemia group compared to non-EBV DNAemia (*P* < 0.05). Gender distribution and type of transplant also showed significant differences between the groups (*P* < 0.05). However, PELD/MELD scores were comparable, with no statistically significant difference (Table 4).

**TABLE 4:** Comparison of clinical parameters between EBV DNAemia and non-EBV DNAemia group

Parameter	EBV DNAemia group (*n* = 50)	Non-EBV DNAemia group (*n* = 207)	Univariate analysis		Multivariate analysis	
			*P*-value	OR (95% CI)	*P*-value	OR (95% CI)
Pre-transplant						
Age in years, median (IQR)	2 (1–5)	36 (11–49)	<0.001	0.94 (0.92–0.96)	0.007	0.96 (0.94–0.99)
Gender						
Male	29 (58%)	162 (78.3%)	0.003	2.60 (1.35–5.00)	0.098	1.94 (0.88–4.27)
Female	21 (42%)	45 (21.7%)				
Type of transplant, *n* (%)						
Living donor liver transplant	49 (98%)	174 (84.1%)	0.009	9.29 (1.24–69.67)	0.124	5.16 (0.63–41.80)
Deceased donor liver transplant	01 (2%)	33 (15.9%)				
MELD/PELD score, median (IQR)	25 (15–30)	25 (18–31)	0.593	0.98 (0.92–1.04)		
EBV risk stratification based on pre-transplant EBV IgG, *n* (%)						
High (D+R−)	28 (56%)	24 (11.6%)	<0.001	9.70 (4.80–19.58)	0.001	4.41 (1.80–10.78)
Intermediate (D+R+)	22 (44%)	183 (88.4%)				
CMV risk stratification based on pre-transplant CMV IgG, *n* (%)						
High (D+R−)	07 (14%)	05 (2.4%)	0.001	6.57 (1.99–21.70)	0.790	1.20 (0.30–4.81)
Intermediate (D+R+)	43 (86%)	202 (97.6%)				
Transplant						
Cold ischemic time in minutes, median (IQR)	58 (45–84)	73 (57–98)	0.072	0.98 (0.97–1.00)		
Warm ischemic time in minutes, median (IQR)	21 (17.5–27)	24 (19–28)	0.085	0.93 (0.87–1.00)		
Duration of surgery in minutes, median (IQR)	660 (597–807)	720 (548–803)	0.788	1.00 (0.99–1.00)		
Blood transfused						
Cases with blood components transfused (up to 5 units)	39 (78%)	142 (68.5%)	0.191	0.61 (0.29-1.27)		
Cases with blood components transfused (>5 units)	11 (22%)	65 (31.5%)				
Post-transplant						
Immunosuppressant details, *n* (%)						
Cases on three drug regime	34 (68%)	129 (62.3%)	0.454	1.28 (0.66–2.47)		
Cases on two drug regime	16 (32%)	78 (37.7%)				
Cases with graft rejection at any point of time post-transplant, *n* (%)	11 (22%)	83 (40.1%)	0.017	0.42 (0.20–0.87)	0.188	0.56 (0.23–1.23)
Cases with CMV infection at any point of time post-transplant, *n* (%)	26 (52%)	74 (35.7%)	0.034	1.94 (1.04–3.63)	0.062	2.12 (0.99–4.55)
Mortality, *n* (%)	06 (12%)	31 (14.9%)	0.590	0.77 (0.30–1.97)		

When transplant factors such as warm ischemia time, cold ischemia time, blood units transfused, and duration of surgery were analyzed, no statistically significant differences were observed between the groups (*P* > 0.05) (Table 4).

Various post-transplant factors were also analyzed and compared between both groups (Table 4). No significant association between immunosuppressive therapy and onset of EBV DNAemia was ascertained (*P* = 0.454). Interestingly, the graft rejection was reported more in the non-EBV DNAemia group compared to the EBV DNAemia group (40.1% vs 22%, *P* = 0.017) which indicates that cases with EBV DNAemia might have a weaker overall immune response, including a reduced ability to mount a robust rejection response. Furthermore, post-transplant CMV infection (>2.7 log10 IU/mL in plasma) was recorded more in the EBV DNAemia group (52%, *n* = 26) compared to the non-EBV DNAemia group (35.7%, *n* = 74), *P* = 0.034. The overall mortality was recorded in 37 cases, with 6 (12%) reported in the EBV DNAemia group and 31 (14.9%) in the non-EBV DNAemia group, *P* = 0.590.

Univariate analysis identified age, gender, type of transplant, pre-transplant EBV/CMV serostatus, graft rejection, and post-transplant CMV infection as factors significantly associated with the development of EBV DNAemia. However, multivariate analysis revealed that only age and pre-transplant EBV serostatus independently predicted post-transplant EBV DNAemia (Table 4). Notably, age below 8 years demonstrated predictive ability for EBV DNAemia, with an AUROC of 0.805, sensitivity of 78.2%, and specificity of 80%.

## DISCUSSION

In the present study, EBV DNAemia was reported in 19.5% of LT recipients, belonging to both high and intermediate-risk groups. DNAemia was predominantly observed in the pediatric population and between 7 and 12 months post-transplantation. To the best of our knowledge, this is the first study from India to determine the occurrence of EBV DNAemia among LT recipients and its association with various clinical parameters across both age groups. In the Indian context, existing studies predominantly focus on pediatric renal transplant recipients, with limited literature on adult LT patients, primarily in the form of case reports (12, 14, 16).

Clinical guidelines recommend EBV DNA monitoring on a weekly to biweekly basis over the first-year post-transplant for all recipients belonging to high-risk (D+/R−). Routine surveillance is not generally advised for R+ (intermediate-risk) SOT recipients, except in intestinal transplants (6). Limited studies had explored the clinical utility of continuous EBV monitoring in SOT recipients, with the majority focusing on the pediatric population due to the high prevalence of D+/R− serostatus. The reported incidence of EBV DNAemia in SOT recipients varies widely (4.1% to 67%) reflecting heterogeneity in study populations, monitoring schedules, EBV DNAemia definitions, detection methods, and cutoff values (1, 2, 5, 16–18). In the present study, EBV DNAemia was observed in 19.5% of LT recipients belonging to both high- and intermediate-risk categories. The incidence of EBV DNAemia may be underestimated due to the infrequent screening conducted in our cohort. The finding highlights the importance of EBV DNAemia monitoring, even in recipients with positive pre-transplant serostatus, especially relevant in India, where EBV seroprevalence is high.

In this study, 58% of cases exhibited EBV DNAemia within the first-year post-transplantation, aligning with findings reported in several other studies (2, 16, 17, 19, 20). While some studies suggest that high-risk recipients tend to exhibit earlier onset of EBV DNAemia along with higher viral loads, others report no such correlation (2, 7, 21, 22). In our cohort, high-risk recipients did show earlier detection of EBV DNAemia; however, there was no significant difference in the viral load between high and intermediate risk categories.

The optimal management of EBV DNAemia post-transplant remains unclear. While RIS is generally considered the mainstay of treatment, the role of antiviral therapy and anti-CD20 agents remains controversial (2, 18, 23, 24). The RIS served as the primary approach to management in our cohort although antiviral therapy was also attempted in a few cases. However, the addition of antiviral did not appear to significantly affect viral kinetics or PTLD outcomes when compared with RIS alone, which aligns with available literature (6).

EBV DNAemia post-transplantation results from the interplay of various clinical factors (2, 5, 19, 22). In this study, clinical factors were further characterized into pre-transplant, transplant, and post-transplant factors and were compared between EBV DNAemia group and non-EBV DNAemia group. Among the pre-transplant factors, age and EBV serostatus were the only variables significantly associated with EBV DNAemia on multivariate analysis. Younger recipients, who often lack prior exposure to EBV, hence seronegative before transplantation, were found to be at a higher risk. This highlights the vulnerability of this population to develop EBV DNAemia due to their immunological naivety (7). Transplant-related factors, such as warm/cold ischemic time, blood loss, and surgery duration are well-established determinants of graft function and overall transplant outcomes. However, their direct influence on EBV DNAemia remains unclear. In our study, no significant association was observed between various transplant factors and the occurrence of EBV DNAemia. Further research is warranted to investigate their potential role in EBV DNAemia.

Post-transplant CMV infection may be associated with increased risk of EBV DNAemia/PTLD due to the interplay of several postulated mechanisms. First, CMV replication is associated with a surge of pro-inflammatory cytokines that promote B-cell activation and proliferation, thereby facilitating EBV-driven lymphoproliferation (18, 25). Second, CMV induces a state of profound immune activation followed by exhaustion, characterized by expansion of CMV-specific CD8^+^ T cells, immunosenescence, and narrowing of the antiviral T-cell repertoire. This skewing may possibly impair EBV-specific cytotoxic T-cell surveillance and permit the uncontrolled expansion of EBV-infected B cells (26). In addition, CMV also exerts direct viral immunomodulatory effects. CMV encodes a viral IL-10 homolog (UL111A) that closely mimics the immunosuppressive activity of host IL-10. This viral cytokine suppresses antigen presentation by downregulating MHC expression and inhibits pro-inflammatory cytokine production, thereby weakening EBV-specific T-cell surveillance (27). Rejection may also contribute to EBV DNAemia through immune activation, cytokine release, and intensified use of immunosuppressive therapy. Immunosuppressants, while critical for preventing graft rejection, suppress immune surveillance mechanisms responsible for controlling latent EBV, thus increasing the risk of viral reactivation and replication. However, data on the association of these factors with increased risk of EBV DNAemia remain inconclusive (1, 2, 5, 18, 28, 29). In our cohort, no association between post-transplant CMV infection, graft rejection, or immunosuppressive therapy and the occurrence of EBV DNAemia was found. These findings suggest that the relationship between clinical factors and EBV DNAemia is complex. Further investigations from India are warranted to improve the understanding of risk factors for EBV DNAemia in transplant recipients.

PTLD is a potentially fatal complication in transplant recipients with reported incidence rate of 1% to 32% in SOT and 3% to 12% following LT (6, 30). EBV is implicated in 70%–80% PTLD cases and is commonly seen within the first 2 years post-transplantation, whereas non-EBV associated PTLD tends to occur later (18). Extremes of age and pre-transplant EBV serostatus of donor/recipients are considered significant risk factors for PTLD (2, 18, 31–33). Although EBV DNAemia is considered a potential risk factor, standardized monitoring time-points, specimen type, and clinically significant cutoff values requiring intervention remain undefined (2, 4, 9, 22, 34). Studies suggest that both the magnitude and duration of EBV DNAemia may influence PTLD risk. Sustained EBV DNAemia was reported in approximately 70% of adult LT recipients, with only a small proportion progressing to PTLD (1, 2, 19, 35, 36). Further research had shown that PTLD occurrence was higher in recipients with chronic high viral load compared to those with low viral load group suggesting both the magnitude and duration of EBV DNAemia are critical factors (10, 37). In the present study, sustained viremia was reported in 71.7% (*n* = 33) recipients among which three cases progressed to EBV-associated PTLD. Clinically, sustained EBV DNAemia may serve as an early warning marker for progression to PTLD. PTLD developed 2 years after transplantation in two cases, exclusively in the pediatric population belonging not only to high-risk but also to intermediate-risk group, consistent with findings reported by others (2, 6, 14, 15, 38). The third case occurred within 1 year of transplantation in a recipient categorized as indeterminate risk. This highlights the need for intensive EBV DNAemia monitoring, regardless of pre-transplant serostatus and a high index of clinical suspicion even beyond 1 year post-transplant, especially in recipients with sustained EBV DNAemia. RIS along with rituximab and/or multi-agent chemotherapy remains the cornerstone of PTLD management, with all cases in this study receiving R-CHOP with RIS (6, 29).

To summarize, our findings showed that EBV DNAemia was detected predominantly among pediatric recipients, with earlier onset and higher viral loads observed in the high-risk group. Notably, PTLD developed beyond the first year post-transplant and occurred in both high- and intermediate-risk groups, preceded by sustained EBV DNAemia without viral clearance. In light of these observations, a risk-adapted approach to EBV DNA monitoring appears particularly relevant in resource-constrained settings such as India, where universal routine PCR surveillance may not be feasible. Under this approach, high-risk pediatric recipients could be monitored more frequently, with testing every three months during the first post-transplant year, whereas those in the intermediate-risk category may undergo testing every 6 months. Once EBV DNAemia is detected, closer follow up is warranted, with the frequency of monitoring individualized according to clinical status and resource availability. Surveillance should extend beyond the first year, as PTLD can develop even after 3 years post-transplant, particularly in cases with sustained DNAemia regardless of risk category. Such a monitoring strategy would enable earlier detection while conserving limited laboratory resources, thereby improving feasibility in low- and middle-income settings. Further multicenter studies from India are warranted to validate these recommendations and optimize risk-adapted surveillance protocols.

This study has several limitations. First, as a single-center retrospective data-based analysis, its findings should be validated through prospective studies with larger and more diverse cohorts. Second, EBV DNA testing was solely initiated at the clinician’s discretion, which may introduce selection bias.

### Conclusion 

In conclusion, this study underscores the clinical importance of EBV DNAemia monitoring in LT recipients, irrespective of pre-transplant serostatus. The findings highlight the need of risk stratification, regular monitoring, and tailored management strategies in mitigating EBV-related complications. While the incidence of PTLD remains low, sustained EBV DNAemia warrants vigilant monitoring, even in intermediate-risk cases, particularly in a high EBV seroprevalence setting like India.
